# High performance layer-by-layer Pt_3_Ni(Pt-skin)-modified Pd/C for the oxygen reduction reaction†
†Electronic supplementary information (ESI) available: TEM images and HRTEM-EDS mapping images. See DOI: 10.1039/c8sc01358f


**DOI:** 10.1039/c8sc01358f

**Published:** 2018-06-26

**Authors:** Jing-Fang Huang, Po-Kai Tseng

**Affiliations:** a Department of Chemistry , National Chung Hsing University , Taichung 402 , Taiwan , Republic of China . Email: jfh@dragon.nchu.edu.tw

## Abstract

Bimetallic Pt–Ni with Pt on the outermost layer and an innermost layer enriched in Ni, referred to as Pt_3_Ni(Pt-skin), is a promising configuration of an electrocatalyst for the oxygen reduction reaction (ORR) in fuel cells. We prepare a core (Pd)/shell (Pt_3_Ni(Pt-skin)) catalyst (Pt_3_Ni(Pt-skin)/Pd/C) from Zn underpotential deposition (UPD) on a Ni UPD modified Pd/C catalyst, facilitating Pt atomic layer-by-layer growth on the Ni surface through the galvanic replacement process. Pt_3_Ni(Pt-skin)/Pd/C shows the best ORR performance, with a Pt specific activity of 16.7 mA cm^–2^ and Pt mass activity of 14.2 A mg_Pt_^–1^, which are 90- and 156- fold improvements over commercial Pt/C catalysts. The Pt_3_Ni(Pt-skin) structure effectively inhibits Ni leaching to improve the durability in two accelerated durability test modes mimicking the catalyst lifetime and start-up/shut-down cycles.

## Abstract 2

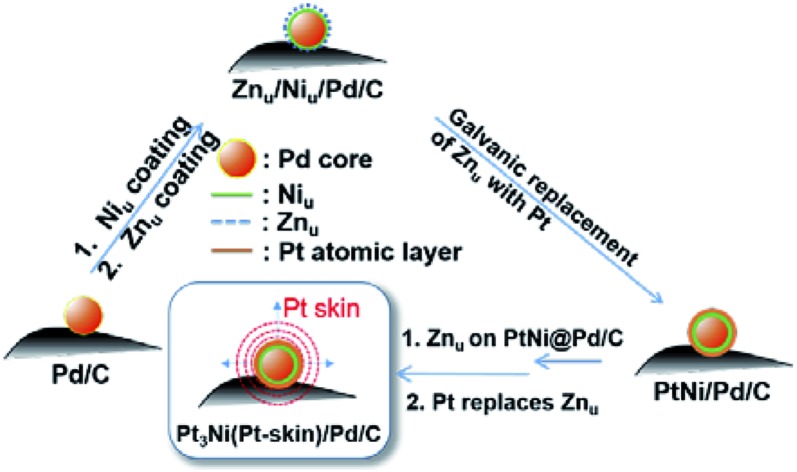
A core (Pd)/shell (Pt_3_Ni(Pt-skin)) ORR catalyst was obtained, and displayed eminently superior catalytic performance to state-of-the-art Pt–Ni catalysts.

## Introduction

Cathodic oxygen reduction reaction ORR electrocatalysts play a crucial role in fuel cell performance.^1^–^3^ The Pt catalyzed ORR has sluggish kinetics and requires a high overpotential, causing lower Pt specific activity (*j*_kPt_, the catalytic activity normalized by the Pt electrochemically active surface area (ECSA)) and Pt mass activity (*i*_mPt_, the catalytic activity per Pt mass). The high cost and the low durability of Pt ORR electrocatalysts remain a challenge for the widespread commercialization of fuel cells.^4^–^18^ Alloying Pt with another transition metal (Co, Ni, Fe, *etc.*) has attracted much attention in the design of advanced electrocatalysts, as this approach not only decreases the Pt content but also enhances the catalytic activity and durability.^8^,^19^–^23^ These designs include core–shell structured nanoparticles (NPs),^24^,^25^ as well as de-alloyed^26^–^28^ and porous structured NPs.^6^,^26^ The Pt alloys may tailor the electronic (affecting the Pt–OH bond energetics) and geometric structures (affecting the Pt–Pt bond distance and coordination number) to enhance the catalytic activity.^8^ The highest recorded *j*_kPt_ values were achieved on single crystal surfaces or well-defined NPs with a specifically engineered facet structure and alloy composition. For example, Stamenkovic *et al.* found that single-crystal Pt_3_Ni (111) with Pt-skin had a *j*_kPt_ value 10 times higher than the corresponding Pt (111) surface and 90 times higher than the commercial Pt/C catalysts used for the ORR.^8^ Pt_3_Ni octahedral NPs were shown to exhibit favorable microstructures for greatly enhanced activity in the ORR,^28^–^30^ but were still limited by their insufficient stability due to Ni leaching from the alloys and decreased ECSA from the agglomeration of the NPs during electrochemical cycling.^31^ Core–shell NPs represent a multi-metallic structure with tunable properties to enhance ORR catalytic activity.^32^–^38^ A promising structure to optimize *i*_mPt_ and Pt utilization is a thin shell or skin layers of Pt or Pt alloys over a non-Pt NP core. As reported by Adzic *et al.*, Cu underpotential deposition (UPD) is used as a sacrificial coating on the core, followed by galvanic replacement (Gal) with noble metal ions for the final shell metal.^7^,^39^–^41^ UPD-Gal is one of the most successful methods to specifically coat Pt monolayers on different metals, but the commonly used Cu UPD (Cu_u_) limits the options of Pt coating substrates due to its high work function, *Φ*_Cu_ (∼4.94 eV).^42^ It is known that when the *Φ* of an electrodeposited metal, *Φ*_M_, is lower than that of the substrate metal, *Φ*_S_, UPD may occur at a potential more positive than the equilibrium potential. The Kolb–Gerischer equation, Δ*E* = 0.5Δ*Φ* (Δ*E* is the underpotential shift in V and Δ*Φ* is *Φ*_M_ – *Φ*_S_ of the electron in eV), has been used to evaluate the level of underpotential shift.^43^ Despite numerous attempts to synthesize Pt alloying nanocatalysts with Pt-skin surfaces on transition metals,^28^,^44^–^50^ it still remains a challenge to demonstrate their existence at the nanoscale. To resolve this issue, we attempted to improve the elegant UPD-Gal approach, also referred to as electrochemical atomic layer deposition (E-ALD) or electrochemical atomic layer epitaxy EC-ALE.^51^,^52^ In UPD-Gal, the UPD adlayer enables a type of surface limited reaction (SLR). SLRs occur only at the substrate or deposit surface and specifically form an atomic layer or a monolayer coverage. The “atomic layer” refers to a coverage less than a monolayer, a monolayer being a unit of deposit coverage. Zn UPD (Zn_u_) was used to replace the Cu_u_ in the UPD-Gal due to its lower value of *Φ*_Zn_ (∼3.95 eV) compared to *Φ*_Cu_,^42^ and the more negative standard reduction potential, *E*^0^ (Zn^2+^/Zn) = –0.76 V.^53^ The lower *Φ*_Zn_ makes Zn_u_ occur on a greater variety of substrates, particularly Ni. Zn_u_ can therefore replace many metals that are nobler than Zn in the Gal process thanks to its more negative *E*^0^.

We report Zn_u_ assisted UPD-Gal (ZnUPD-Gal) in a Ni UPD (Ni_u_) process of constructing a layer-by-layer Pt_3_Ni(Pt-skin) thin layer on a carbon-supported Pd electrocatalyst (Pd_20_/C) (20 wt% Pd on XC-72 Valcan carbon, E-TEK) (Pt_3_Ni(Pt-skin)/Pd_20_/C). The Pt_3_Ni(Pt-skin) structure retains the advantages of the ultra-thin layer structure and the synergetic effects of the Ni sublayer. Pt_3_Ni(Pt-skin)/Pd_20_/C possesses an ultra-high *j*_kPt_ = 16.7 mA cm^–2^ and *i*_mPt_ = 14.2 A mg_Pt_^–1^ (Pt loading = 2.97 μg_Pt_ cm^–2^), at 0.9 V *vs.* RHE, which are 90-fold and 156-fold improvements, respectively, over commercial Pt/C catalysts (0.185 mA cm^–2^ and 0.091 A mg_Pt_^–1^, Pt loading = 24 μg_Pt_ cm^–2^). We also show that the perfect Pt_3_Ni(Pt-skin) structure effectively inhibits Ni leaching, significantly improving the durability of catalysts.

## Results and discussion

**Fig. 1:**
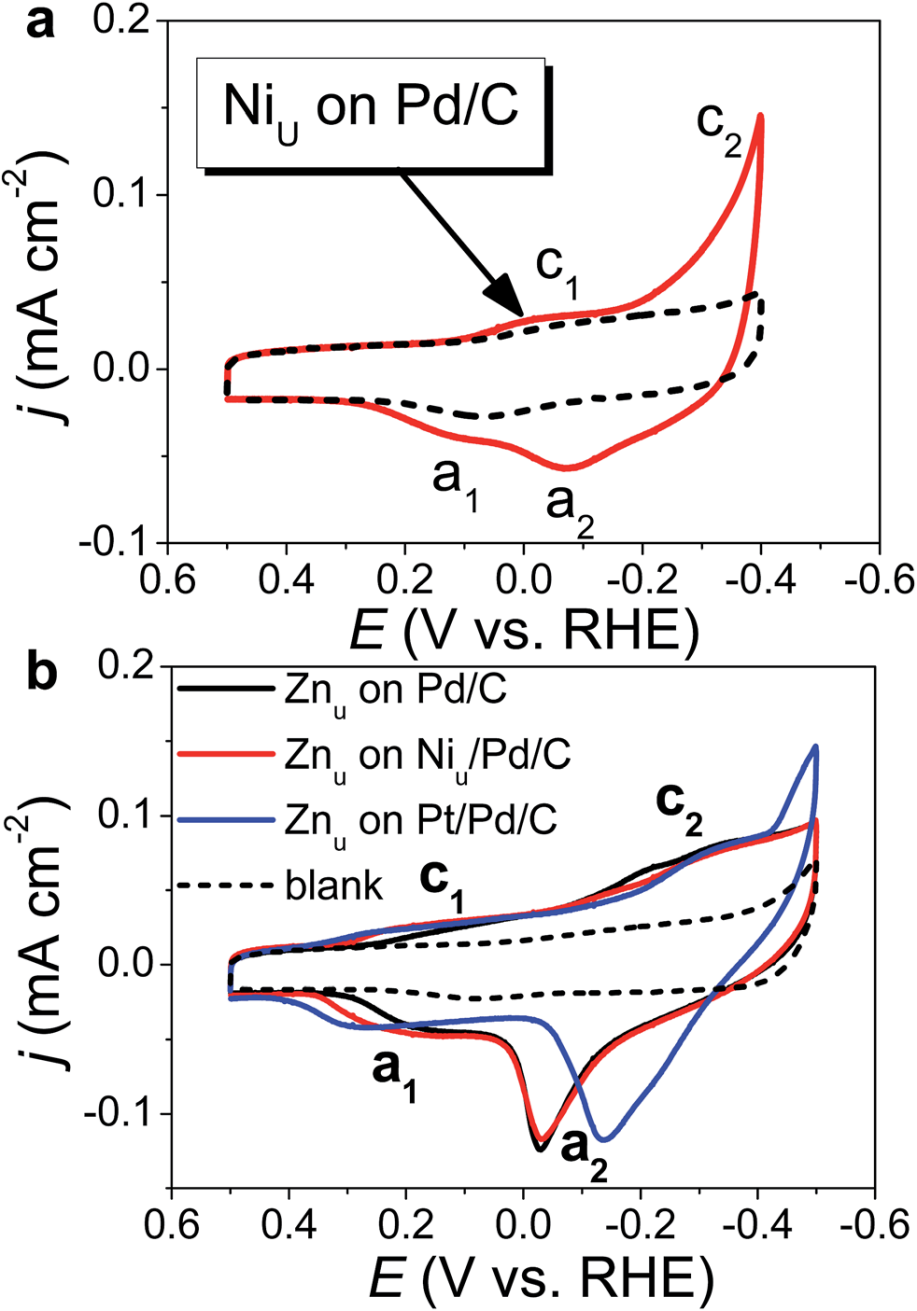
CVs of (a) Ni_u_ on Pd_20_/C@GC and (b) Zn_u_ on Pd_20_/C@GC, Ni_u_/Pd_20_/C@GC, and Ni_u_/Pd_20_/C@GC recorded in Ar saturated 0.5 M Na_2_SO_4aq_ (solid line) with and (dashed line) without (a) 20 mM NiSO_4_ and (b) 20 mM Zn(ClO_4_)_2_ at a sweep rate of 50 mV s^–1^.

Fig. 1 shows the CVs of Ni_u_ and Zn_u_ on Pd_20_/C@GC in an Ar-saturated 0.5 M Na_2_SO_4_ aqueous solution (Na_2_SO_4aq_). These voltammograms both show two pairs of redox waves, c_1_/a_1_ and c_2_/a_2_, which correspond to UPD/stripping and bulk deposition (OPD)/bulk stripping, respectively. These indicate that Ni_u_ or Zn_u_ can modify the Pd surface through controlled potential electrodeposition. Herein Zn_u_ was used to assist in the selective growth of Pt atomic layers on a given metallic surface, here Ni and Pt. Zn_u_ was further studied on Ni_u_/Pd_20_/C@GC and Pt atomic layer covered Pd_20_/C@GC (Pt/Pd_20_/C@GC) (Fig. 1b). Ni_u_/Pd_20_/C was from direct electrodeposition of Ni_u_ on Pd_20_/C in 0.5 M Na_2_SO_4aq_ containing 20 mM NiSO_4_. Pt/Pd_20_/C was prepared by UPD-Gal to deposit a Pt atomic layer on Pd_20_/C. The Zn_u_ redox waves, c_1_/a_1_, on Pd_20_/C positively shifted from ∼0.18 V to ∼0.2 V and ∼0.3 V after the modification of the Ni_u_ and Pt layer, respectively. The changes in CVs are ascribed to Zn_u_ and are surface dependent. Although Zn_u_ on Pt has been reported in the literature,^54^ Zn_u_ on Ni has not been observed until now as the Ni_u_ was freshly produced without serious oxide or hydroxide surface inhibitors. The Zn_u_ on Pd, Ni, and Pt surfaces is related to the higher *Φ*_S_ of these substrates, *Φ*_Pd_ (∼5.0 eV), *Φ*_Ni_ (∼4.91 eV) and *Φ*_Pt_ (∼5.4 eV), in comparison with the lower *Φ*_Zn_ (∼3.95 eV).^42^ Based on the Kolb–Gerischer equation, we could approximately calculate Δ*E* for the metal couples, Pd substrate/Zn, Ni substrate/Zn and Pt substrate/Zn, as ∼0.53 V, ∼0.48 V and ∼0.7 V, respectively. This provides grounds for realizing that Zn_u_ on Pd, Ni and Pt is possible. In comparison, Cu_u_ cannot occur on Ni surfaces due to *Φ*_Cu_ (∼4.94 eV) being close to *Φ*_Ni_. This implies that Zn_u_ is a more suitable candidate to promote the Pt atomic layer to specifically form on a Ni surface.

**Scheme 1:**
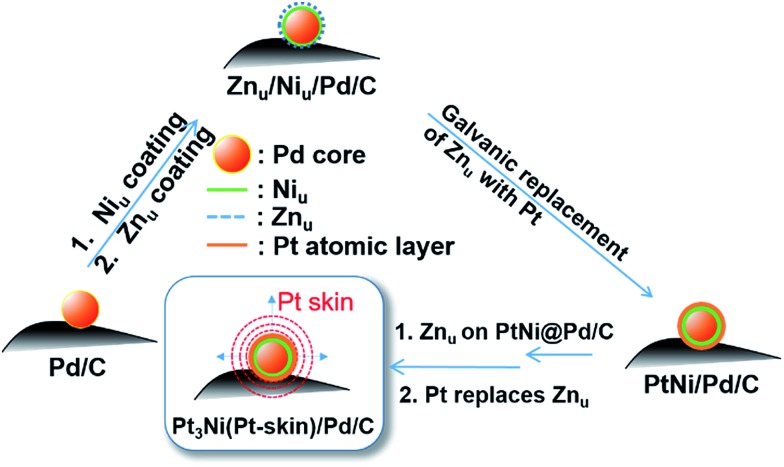
The preparation process of Pt_3_Ni(Pt-skin)/Pd_20_/C electrocatalysts through ZnUPD-Gal on Ni_u_.

**Fig. 2:**
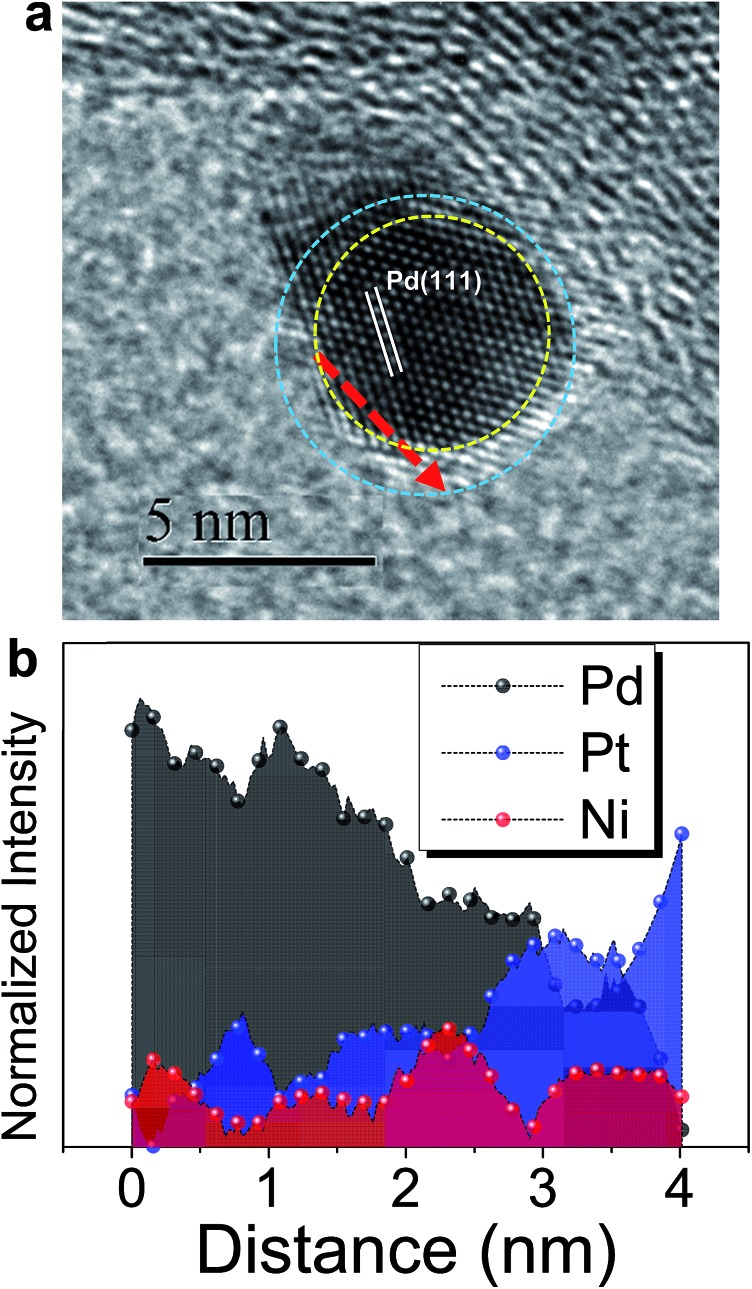
(a) The representative HRTEM image of a core (Pd)/shell (Pt_3_Ni(Pt-skin)) nanoparticle; (b) the corresponding EDS line-scan profile along the red dashed line as shown in (a).

Based on the findings of Zn_u_ on Ni, Pd and Pt surfaces and Ni_u_ on Pd and Pt surfaces, ZnUPD-Gal was used to prepare a Pt_3_Ni(Pt-skin)/Pd_20_/C electrocatalyst (Scheme 1). First, Ni_u_ was electrodeposited on the Pd surface of Pd_20_/C, followed by modification of Zn_u_ on Ni_u_/Pd_20_/C. Subsequently, Zn_u_ was replaced by Pt in the Gal process, leading to Pt atomic layer covered Ni_u_/Pd_20_/C (PtNi/Pd_20_/C). The repetitive ZnUPD-Gal continually introduced the second and the third Pt atomic layers onto PtNi/Pd_20_/C to obtain the desired electrocatalyst, Pt_3_Ni(Pt-skin)/Pd_20_/C. The micromorphology and elemental composition distribution of Pt_3_Ni(Pt-skin)/Pd_20_/C were examined by high-resolution transmission electron microscopy (HRTEM) combined with energy dispersive X-ray spectroscopy (EDS) (Fig. 2a and b and S1†). In Fig. S1a–c,† typical bright-field TEM images of pristine Pd_20_/C reveal a relatively uniform dispersion of Pd NP (Pd_nano_) with sizes of approximately 4.8–5.6 nm (diameter). After Pt_3_Ni(Pt-skin) modification, the growth of the particle size occurred as expected (diameter increased to ∼6.8 nm) (Fig. S1d–f†). The core of Pt_3_Ni(Pt-skin)/Pd_nano_ shows clear fringe orientations of the Pd (111) single-crystal structure (Fig. 2a). The EDS line profile analysis shows the distribution of Pt, Ni, and Pd components in a single NP (Fig. 2b). The width of Pt_3_Ni(Pt-skin)/Pd_nano_ examined was 4 nm, as designated by the red line in Fig. 2a. The line profile analysis validates the core–shell structure, which is a Pd core covered by an ultra-thin PtNi shell with a thickness of approximately 1.2 nm. The Pd composition is constant and high in the particle center, and decreases from the edge of the core to the particle surface. Most of the Ni component is located at the interface between the Pd core and PtNi shell. At the exterior of the PtNi shell, the Pt intensity is approximately 2.5 times that of Ni in the interior of the PtNi shell. This analysis demonstrates the formation of a core (Pd)/shell (Pt_3_Ni(Pt-skin)) structure. To extend the diversity of the ZnUPD-Gal with the Ni_u_ process, a three repeated PtNi layer covered Pd core ((PtNi)_3_/Pd_20_/C) was also prepared as a control example. The EDS line profile analysis of (PtNi)_3_/Pd_20_/C confirms that a Pd core/(PtNi)_3_ shell structure was successfully obtained using this process (Fig. S2 and S3†).

**Fig. 3:**
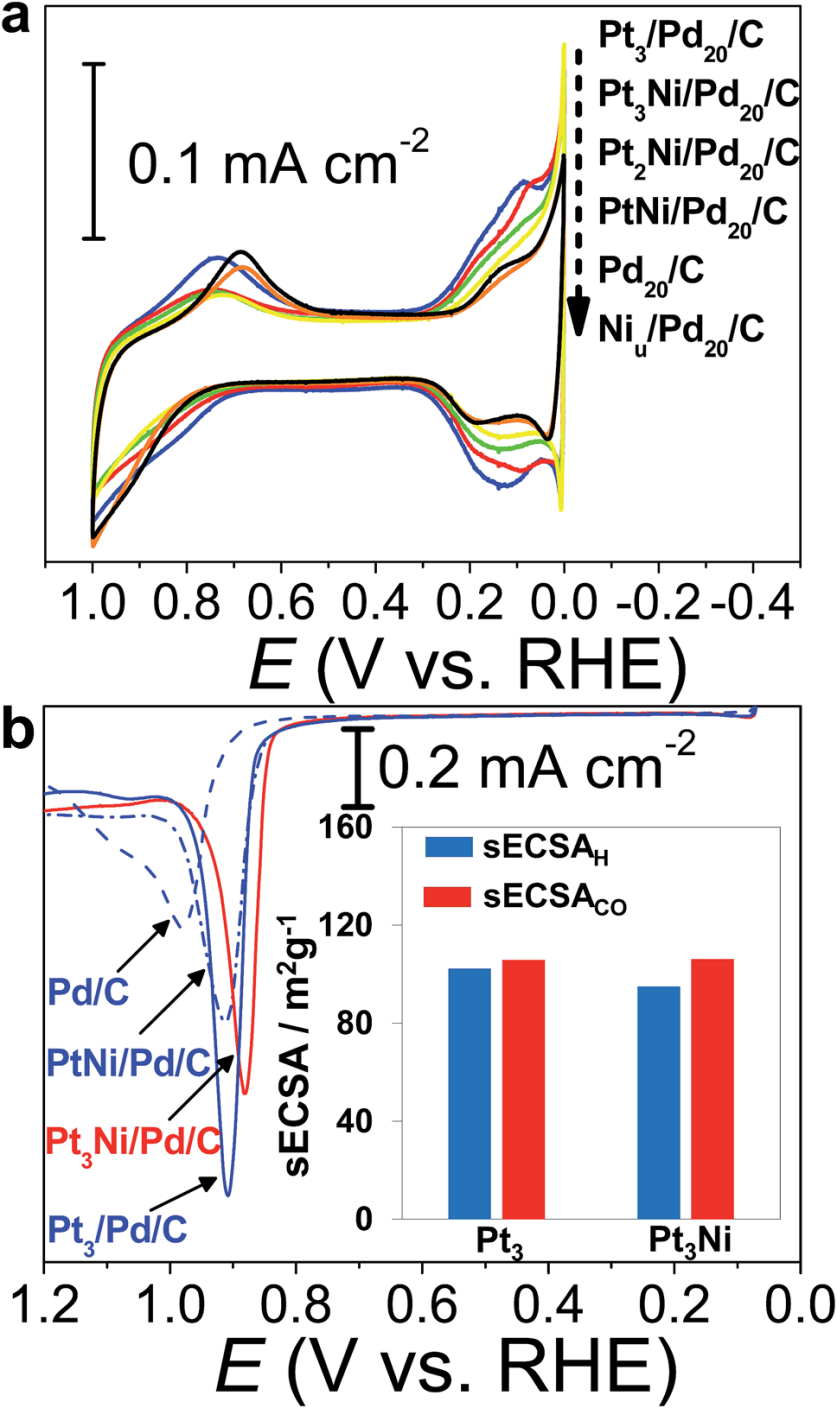
Electrochemical surface characterization of electrocatalysts by using a GC electrode in 0.1 M H_2_SO_4aq_: (a) cyclic voltammograms of (blue) Pt_3_/Pd_20_/C, (red) Pt_3_Ni(Pt-skin)/Pd_20_/C, (green) Pt_2_Ni(Pt-skin)/Pd_20_/C, (yellow) PtNi(Pt-skin)/Pd_20_/C, (black) Pd_20_/C, and (orange) Ni_u_/Pd_20_/C; (b) CO stripping curves. The inset shows (blue) sECSA_H_ and (red) sECSA_CO_ for (Pt_3_) Pt_3_/Pd_20_/C and (Pt_3_Ni) Pt_3_Ni(Pt-skin)/Pd_20_/C.

The CVs of H_2_SO_4aq_ were used to track different modified layers grown on the Pd surface. The typical CV of Pd_20_/C shows redox peaks corresponding to the formation and removal of Pd hydroxide (Pd(OH)_*x*_) (0.6–1.0 V *vs.* RHE) and H absorption (H_ab_)/desorption (0.3–0.0 V *vs.* RHE) (Fig. 3a). In comparison with the CV of pristine Pd_20_/C, the first modified layer, Ni_u_, on Pd_20_/C inhibits the formation of Pd(OH)_*x*_ and H_ab_, causing the reduction of related redox charges on Ni_u_/Pd_20_/C. The following Pt atomic layers were grown in turn on the Ni_u_ surface by ZnUPD-Gal. With the increase of the Pt layer, characteristic Pt redox waves, including the formation of Pt hydroxide (Pt(OH)_*x*_) occurred at a more negative potential (∼0.75 V *vs.* RHE) and hydrogen adsorption (H_ad_)/desorption waves gradually grew. For the purpose of comparison, three Pt layer-covered Pd_20_/C@GC (Pt_3_/Pd_20_/C@GC) without Ni_u_ was also prepared using UPD-Gal. This indicated that the surface of Pt_3_/Pd_20_/C was mainly composed of Pt, due to the similar CV features of Pt_3_/Pd_20_/C and Pt/C. In comparison to the CV features of Pt_3_/Pd_20_/C, those of Pt_3_Ni(Pt-skin)/Pd_20_/C based on the same Pt content showed that the onset potential of H_ad_ shifted towards a more negative potential and the formation of Pt(OH)_*x*_ occurred at a more positive potential. This is a consequence of the electronically modified structure of Pt for “Pt skin” surfaces by the subsurface Ni atoms, which leads to weakened interactions between Pt and adsorbates such as H_ad_ and surface hydroxides (OH_ad_). This is also typical for the Pt_3_Ni(Pt-skin) structure.^8^,^55^ The H_ad_ integrated charge is a conventional approach in the estimation of ECSA (ECSA_H_).^56^ However, the suppression of H_ad_ on the Pt_3_Ni(Pt-skin) structure can substantially affect the accurate estimation of the real ECSA. The electro-oxidation of adsorbed carbon monoxide (CO_ad_), known as CO stripping, has been suggested as a complementary ECSA evaluation method (ECSA_CO_).^55^,^57^ Fig. 3b shows the voltammetric curves of CO stripping obtained for pristine Pd_20_/C, PtNi/Pd_20_/C, and Pt_3_Ni(Pt-skin)/Pd_20_/C. The oxidation of the CO_ad_ takes place in a single peak whose peak potential shifts towards more negative values as the Pt content increases. The broad CO stripping peak for Pd_20_/C becomes sharper with increasing Pt content in the PtNi shell.^58^ These results suggest that the PtNi shell significantly weakens the interaction of Pd surface atoms with CO_ad_. Interestingly, the onset of CO stripping on Pt_3_Ni(Pt-skin)/Pd_20_/C is more negatively shifted than on Pt_3_/Pd/C, and the shape of the stripping peak is broader due to the weaker interaction of the Pt surface atoms with CO from the Ni sublayer. However, the similar charge of CO oxidation points to an equal coverage of CO. The specific ECSA (sECSA = ECSA/metal loading, m^2^ g^–1^) for Pt_3_Ni/Pd/C and Pt_3_/Pd/C electrocatalysts was evaluated from ECSA_H_ (sECSA_H_) and ECSA_CO_ (sECSA_CO_), respectively (Fig. 3b). Although the suppression of sECSA_H_ on Pt_3_Ni/Pd/C (95.1 m^2^ g^–1^) was observed in comparison with that of Pt_3_/Pd/C (102.3 m^2^ g^–1^), sECSA_CO_ shows a similar value of ∼106 m^2^ g^–1^ on both Pt_3_Ni(Pt-skin)/Pd/C and Pt_3_/Pd/C.

**Fig. 4:**
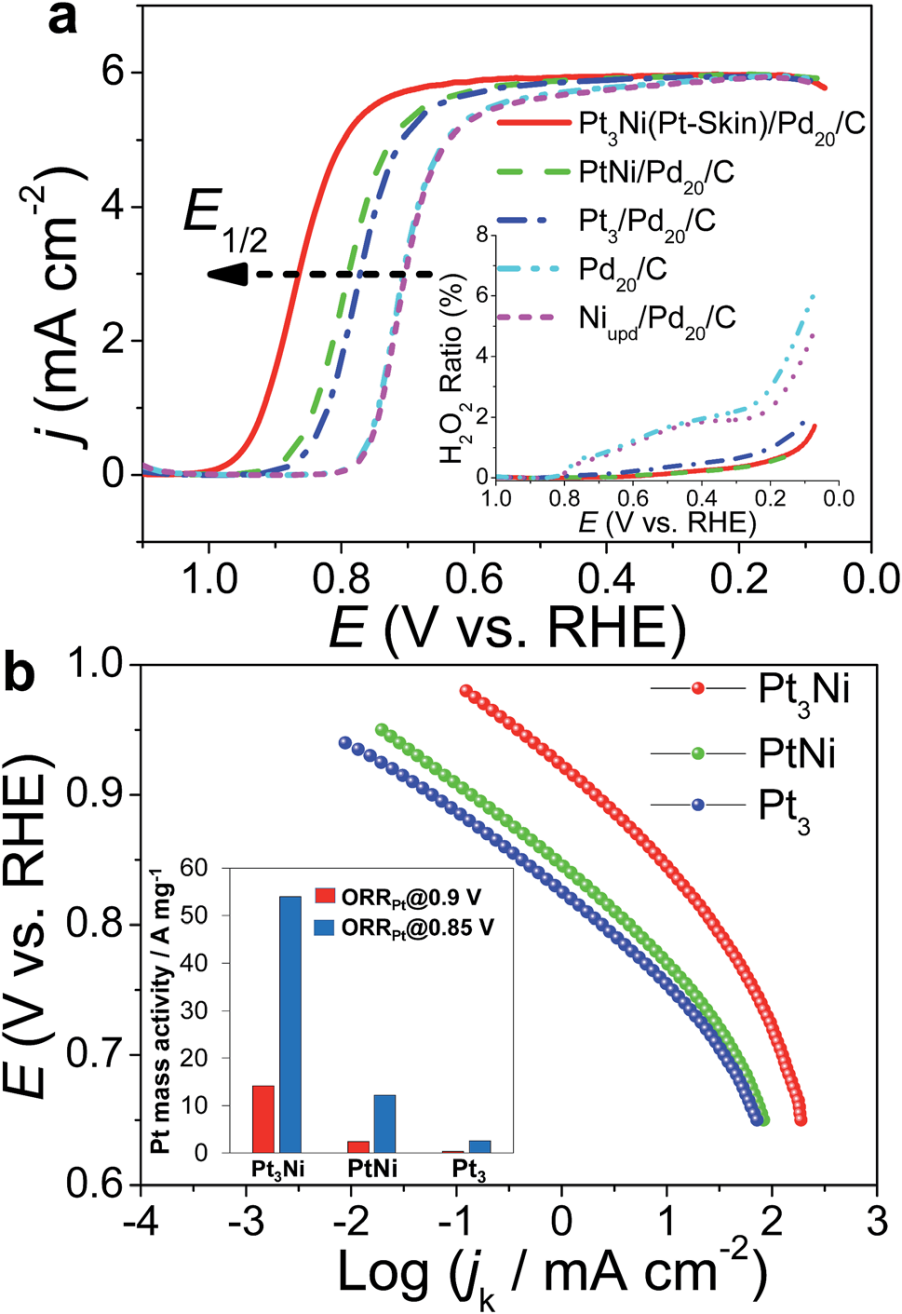
(a) ORR polarization curves of Pt_3_Ni(Pt-skin)/Pd_20_/C, PtNi/Pd_20_/C, Pt_3_/Pd_20_/C, Ni_u_/Pd_20_/C, and commercial Pd_20_/C catalysts recorded at room temperature in an O_2_-saturated 0.1 M HClO_4_ aqueous solution with a scan rate of 10 mV s^–1^ and a rotation rate of 1600 rpm. Inset: the corresponding H_2_O_2_ production current efficiency, *χ*_H_2_O_2__, from ring current (*i*_r_) during the ORR, ring potential = 1.1 V, collection efficiency: *N* = 0.2. (b) The corresponding Tafel plots. Inset: (ORR@0.85 V and ORR@0.9 V) mass activities and (ORR_Pt_@0.85 V and ORR_Pt_@0.9 V) Pt mass activities for (Pt_3_Ni) Pt_3_Ni(Pt-skin)/Pd_20_/C, (PtNi) PtNi/Pd_20_/C, and (Pt_3_) Pt_3_/Pd_20_/C measured at 0.85 V and 0.9 V.

The ORR polarization curves were obtained with Pd_20_/C, Ni/Pd_20_/C, Pt_3_/Pd_20_/C, PtNi/Pd_20_/C and Pt_3_Ni(Pt-skin)/Pd_20_/C electrocatalysts as thin films on the GC disc electrode of a RRDE in an O_2_-saturated 0.1 M HClO_4_ solution at 1600 rpm (Fig. 4a). The Pt ring electrode of the RRDE was potentiostated at 1.1 V to collect the ring current (*i*_r_) related to the H_2_O_2_ oxidation reaction. The polarization curves on the disc electrode displayed two distinguishable potential regions: well-defined diffusion limiting currents (*i*_D_) for the ORR below 0.7 V and a mixed kinetic-diffusion control region between 0.7 and 1.1 V. In both potential regions, *i*_r_ was a rather small fraction of *i*_D_ for all electrocatalysts, revealing that the ORR proceeds almost entirely through the 4e^–^ reduction pathway. A quantitative presentation of the H_2_O_2_ production (current efficiency, *χ*_H_2_O_2__) was given using eqn (1):^20^1
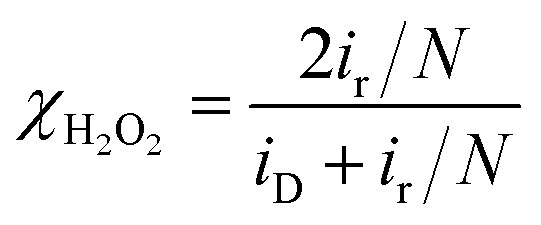
where *N* is the collection efficiency of the RRDE. In the potential region of 0.05 < *E* < 1 V, similarly small amounts of H_2_O_2_ were detected on the ring electrode from the Pd_20_/C and Ni_u_/Pd_20_/C electrocatalysts, implying that Ni modification does not alter the reaction pathways. Pt-containing shells on the other three electrocatalysts (Pt_3_/Pd_20_/C, PtNi/Pd_20_/C and Pt_3_Ni(Pt-skin)/Pd_20_/C) effectively inhibited the production of H_2_O_2_ during the ORR, since there was no detectable H_2_O_2_ on the ring electrode in the kinetically controlled potential region, implying that Pt atomic layers perfectly covered the outermost layer of Pt-containing shells. The half-wave potential of an ORR polarization curve, *E*_1/2_, is often used to evaluate the electrocatalytic activity of a catalyst. *E*_1/2_ increased in the following sequence: Pd_20_/C ∼ Ni_u_/Pd_20_/C < Pt_3_/Pd_20_/C < PtNi/Pd_20_/C ≪ Pt_3_Ni(Pt-skin)/Pd_20_/C. Pt_3_Ni(Pt-skin)/Pd_20_/C showed a marked positive shift in *E*_1/2_ of 200 mV and 100 mV relative to Pd_20_/C and Pt_3_/Pd_20_/C, respectively. These data show that the Pt_3_Ni(Pt-skin) structure exhibits marked activity improvements over Pd_20_/C and Pt_3_/Pd_20_/C catalysts. Fig. 4b compares the Tafel plots for the specific activity (*j*_k_) towards the ORR obtained by normalizing the kinetic current (*i*_k_) to the ECSA_CO_ for Pt_3_/Pd_20_/C, PtNi/Pd_20_/C and Pt_3_Ni(Pt-skin)/Pd_20_/C, at 1600 rpm in the cathodic sweep direction. *i*_k_ was obtained from the measured currents, corrected for mass transport according to eqn (2).^59^–^61^
2
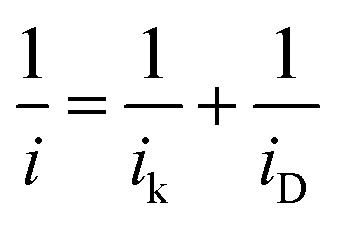



**Fig. 5:**
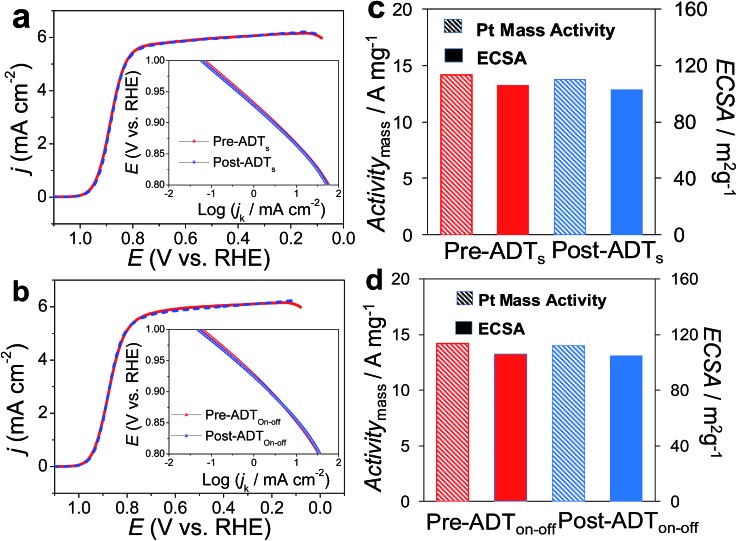
ORR polarization curves of the Pt_3_Ni(Pt-skin)/Pd_20_/C catalyst on a GC RDE (0.196 cm^2^, ∼2.97 μg cm^–2^ Pt loading) (dashed line) before and (solid line) after accelerated durability tests include (a) ADT_s_: a linear potential polarization from 0.6 to 1.0 V *vs.* RHE at a scan rate of 50 mV s^–1^ for 20 000 cycles and (b) ADT_on–off_: a linear potential polarization from 1.0 to 1.5 V *vs.* RHE for 20 000 cycles at a scan rate of 500 mV s^–1^ in O_2_-saturated 0.1 M HClO_4_. Insets: the corresponding Tafel plots. (c) and (d) (solid pattern) sECSA_CO_, and (slash pattern) Pt mass activities for (blue) before and (red) after accelerated durability tests.

Two Tafel slopes of Pt_3_/Pd_20_/C, –60/–119 mV dec^–1^, were similar to those observed on conventional Pt/C. The values of the Tafel slopes of Pt_3_Ni(Pt-skin)/Pd_20_/C, –70/–122 mV dec^–1^, showed a slight difference from those of Pt_3_/Pd_20_/C, probably owing to a different state and coverage of Pt(OH)_*x*_ after the insertion of the Ni sublayer. When comparing *j*_k_ for different electrocatalysts at 0.9 V, the specific activity of Pt_3_Ni(Pt-skin)/Pd_20_/C was 2.34 mA cm^–2^, which is much higher than those of Pt_3_/Pd_20_/C (∼0.06 mA cm^–2^) and PtNi/Pd_20_/C (∼0.16 mA cm^–2^). Considering the ECSA_CO_ contribution from Pt (ECSA_CO(Pt)_ = sECSA_CO_ × the mass of Pt (∼2.97 μg_Pt_ cm^–2^)), Pt_3_Ni(Pt-skin)/Pd_20_/C shows a very high specific activity *j*_k_ of Pt (*j*_kPt_) 16.7 mA cm^–2^ at 0.9 V, which is 90-fold enhancement over commercial Pt/C catalysts (0.185 mA cm^–2^, Pt loading = 24 μg cm^–2^) and even higher than the possibly highest record, 10.3 mA cm^–2^, for octahedral Mo–Pt_3_Ni/C (the particle shape is close to spherical)^62^ and 11.5 mA cm^–2^, for jagged Pt nanowires from de-alloying PtNi nanowires with a totally different microstructure.^31^ The Pt content was obtained by measuring the charge associated with Zn_u_ (after correcting for the double layer charging) on Pd_20_/C and assuming that there was a one-to-one ratio between the UPD adlayer and the Pt atoms. The total amount of Pt on Pt_3_/Pd_20_/C and Pt_3_Ni(Pt-skin)/Pd_20_/C (∼2.97 μg_Pt_ cm^–2^) was much less than that on the commercial Pt/C (∼24 μg_Pt_ cm^–2^). This finding highlights the advantages of ultra-thin layer electrocatalysts, which contribute to not only the reduction of Pt content but also the enhancement of catalytic activity thanks to their high Pt utilization.^39^,^40^ The Pt mass activities (*i*_mPt_) at 0.9 and 0.85 V were calculated based on the ORR polarization curves and the amount of Pt on the electrodes. The Pt_3_Ni(Pt-skin) structure retained the advantages of the ultra-thin layer structure and the synergetic effect of the Ni sublayer presented an ultra-high *i*_mPt_ of 14.2 A mg_Pt_^–1^ based on Pt loading (2.97 μg_Pt_ cm^–2^) at 0.9 V. The *i*_mPt_ of the Pt_3_Ni(Pt-skin)/Pd_20_/C catalyst achieved a 37- and 156-fold improvement over Pt_3_/Pd_20_/C (0.381 A mg_Pt_^–1^, ∼2.97 μg_Pt_ cm^–2^) and commercial Pt/C catalysts (0.091 A mg_Pt_^–1^, ∼24 μg_Pt_ cm^–2^), respectively. The *i*_mPt_ is more than an order of magnitude greater than the U.S. Department of Energy's 2017 goal (0.44 A mg_Pt_^–1^). The *i*_mPt_ of Pt_3_Ni(Pt-skin)/Pd_20_/C is even higher than those of the state-of-the-art Pt–Ni catalysts, including the recently reported PtNi nanoframe catalysts (5.7 A mg_Pt_^–1^),^28^ Mo–Pt_3_Ni/C (6.98 A mg_Pt_^–1^, Pt loading = 4.08 μg_Pt_ cm^–2^)^62^ and jagged Pt nanowires (13.6 A mg_Pt_^–1^, Pt loading = 2.2 μg_Pt_ cm^–2^) (Table 1).^31^ Two accelerated durability test (ADT) modes further examined the Pt_3_Ni(Pt-skin)/Pd_20_/C lifetime (Fig. 5).

**Table 1:** Performance of the Pt_3_Ni(Pt-skin)/Pd_20_/C catalyst and some representative results with high performance from recent published studies^*a*^

Catalysts	Pt loading (μg cm^–2^)	sECSA_CO_ (m^2^ g^–1^)	*j*_kPt_ (mA cm^–2^)	*i*_mPt_ (A mg_Pt_^–1^)	
Pt_3_Ni(Pt-skin)/Pd_20_/C	2.97	106	16.7	14.2	This work
Mo–Pt_3_Ni/C	4.08	83.9	8.2	6.98	Ref. 62
Pt_3_Ni/C nanoframes	N/A	N/A	1.48	5.7	Ref. 28
Jagged Pt nanowires	2.2	118	11.5	13.6	Ref. 31
Commercial Pt/C	24	91	0.185	0.091	This work

^*a*^N/A: not available.

One was a commonly used test mode (ADT_s_) which applied a linear potential sweep from 0.6 to 1.0 V *vs.* RHE at a scan rate of 50 mV s^–1^ for 20 000 cycles in O_2_-saturated 0.1 M HClO_4_. Recently, another ADT mode (ADT_on–off_), start-up/shut-down cycles, has gradually received attention.^63^,^64^ The ADT_on–off_ performed a linear potential sweep from 1.0 to 1.5 V *vs.* RHE for 20 000 cycles at a scan rate of 500 mV s^–1^ in an O_2_-saturated 0.1 M HClO_4_. After ADT_s_, Pt_3_Ni(Pt-skin)/Pd_20_/C retained its ECSA and high activity (Fig. 5a and c), exhibiting only an ∼2 mV shift for its *E*_1/2_. This result is also consistent with the pioneering work by Adzic's group in which the modification of Pt_nano_ surfaces with Au clusters through UPD-Gal improved the stability of catalysts.^7^ Our previous studies also demonstrated that nonspecific noble metal cluster (Pt, Pd or Au) modification through UPD-Gal enhanced the interaction between Pt_nano_ and the carbon support to improve catalyst durability during ADT_s_.^11^ Interestingly, Pt_3_Ni(Pt-skin)/Pd_20_/C shows the highest durability during ADT_on–off_ (Fig. 5b and d). In the ADT_on–off_, Pt dissolution and Ni leaching were serious issues for typical Pt/C or PtNi catalysts owing to their operation at a higher anodic potential. The superior stability of Pt_3_Ni(Pt-skin)/Pd_20_/C during the ADT_on–off_ may be a result of the perfect Pt_3_Ni(Pt-skin) structure. The Pt-skin located on the exterior of the PtNi shell effectively inhibits the dissolution of the Pd core and interior Ni sublayer leaching. The issue of Pt dissolution may have been improved due to the enlargement of the particle sizes of Pt_3_Ni(Pt-skin)/Pd_nano_ in comparison with pristine Pd_nano_ (from ∼5 nm to ∼6.8 nm). It has previously been demonstrated that the Pt dissolution is significantly dependent on particle size and environmental acidity.^65^ A larger particle size (>5 nm) could both reduce the trend of Pt dissolution and its activity. However, the ultra-thin layer Pt_3_Ni(Pt-skin) modification not only stabilizes Pt from particle size enlargement but also significantly enhances catalytic activity owing to the full exposure of Pt active sites on the surface of Pd/C.

## Conclusions

In conclusion, considering that the Pt_3_Ni(Pt-skin) structure exhibited the highest catalytic activity ever recorded, in this work, the challenge of preparing a nanocatalyst mimicking the Pt_3_Ni(Pt-skin) structure was mitigated by growing a Ni_u_ covered Pd_nano_ core using the ZnUPD-Gal procedure. ZnUPD-Gal is an improvement to common CuUPD-Gal that offers more options of coating surfaces and facilitates more metal growth in the Gal process owing to the intrinsic lower *Φ*_Zn_ and more negative *E*^0^ (Zn^2+^/Zn). The Pt_3_Ni(Pt-skin) thin layer with ultra-low Pt loading presents not only a breakthrough in Pt specific activity and Pt mass activity, both of which were superior to those of state-of-the-art Pt–Ni catalysts, but also in high durability owing to the perfect Pt_3_Ni(Pt-skin) construction.

## Experimental

### Chemicals

Carbon-supported palladium (Pd_20_/C) (20 wt% Pd on XC-72 Valcan carbon (E-TEK)), Pt_20_/C (20 wt% Pt on XC-72 Valcan carbon (E-TEK)), 70% HClO_4_ (JT-Baker), 95–98% H_2_SO_4_ (Aldrich), 98% NiSO_4_·6H_2_O (Aldrich), 98% Zn(ClO_4_)_2_·6H_2_O (Aldrich), 99.7% Cu(NO_3_)_2_ (JT-Baker), 99.9% K_2_PtCl_4_ (Alfa), and 5% Nafion (NF) perfluorinated resin in a mixture of lower aliphatic alcohols and water (Aldrich) were used as received.

### Electrochemical experiments

The electrochemical experiments were conducted using a CHI 760C potentiostat/galvanostat and a three-electrode electrochemical cell. To prevent Cl^–^ interference from the typical Ag/AgCl reference electrode, Hg/HgSO_4_ (0.5 M H_2_SO_4_) was used as the reference electrode and Pt wire was used as the counter electrode. The potentials were measured against the reference electrode and converted to the reversible hydrogen electrode (RHE) reference scale by *E*_RHE_ = *E*_Hg/HgSO_4__ + 0.68 + 0.059pH_electrolyte_. All potentials in this paper were stated with reference to the RHE. A glassy carbon (GC) electrode (Pine, 5.0 mm diameter, 0.196 cm^2^ for the fabrication of electrocatalysts or the examination of electrocatalyst performance for the ORR) served as the substrate electrode for the Pd_20_/C electrocatalyst suspension. The Pd_20_/C catalyst suspension was prepared by mixing 10 mg of commercial Pd_20_/C electrocatalyst powder in 5 mL of deionized water (with specific resistivity = 18.2 MΩ cm), followed by the gradual addition of 1 ml of isopropyl alcohol and 20 min of ultrasonication to obtain a Pd_20_/C suspension. The Pd_20_/C@GC electrode was fabricated using a drop coating procedure. Briefly, a GC electrode was polished successively with 1.0, 0.3, and 0.05 μm alumina powder cloth (Buchler) followed by sonication in deionized water and drying prior to use. 2–7 μl of the Pd_20_/C suspension was pipetted onto the surface of the GC electrode as a circle with a geometric area of 0.01–0.05 cm^2^. Pd_20_/C@GC was obtained after drying under an Ar flow at room temperature (∼28 °C) for solvent evaporation.

### Preparation of Ni_u_/Pd_20_/C, PtNi/Pd_20_/C, Pt_3_Ni(Pt-skin)/Pd_20_/C, Pt_3_/Pd_20_/C, and (PtNi)_3_/Pd_20_/C electrocatalysts

Pd_20_/C@GCE was used as a working electrode to prepare relative catalysts. The microstructure of the post-modified Pd_20_/C electrocatalysts was examined by the electrochemical characterization and image analysis. High-resolution transmission electron microscopy (TEM) images (HRTEM) and elemental composition micro-distribution of post-modified Pd_20_/C electrocatalysts were obtained using a JEOL JEM-2100F field emission TEM (FE-TEM) equipped with an energy dispersive X-ray spectrometer (EDS).

#### Preparation of Ni_u_/Pd_20_/C

The Ni underpotential deposition (Ni_u_) covered Pd_20_/C@GC (Ni_u_/Pd_20_/C@GC) electrocatalyst was prepared by direct electrodeposition of Ni_u_ on the Pd surface of the Pd_20_/C@GC electrode from a 0.5 M Na_2_SO_4_ aqueous solution (Na_2_SO_4aq_) containing 20 mM NiSO_4_. The potential was stopped at –0.1 V *vs.* RHE for 20 min to allow the Ni_u_ to completely cover the entire Pd surface. All operations were carried out in a three-electrode electrochemical cell under an Ar atmosphere.

#### Preparation of PtNi/Pd_20_/C

The Pt atomic layer was prepared by ZnUPD-Gal on the Ni surface of the as-prepared Ni_u_/Pd_20_/C@GC from 0.5 M Na_2_SO_4aq_ containing 20 mM Zn(ClO_4_)_2_. The potential was stopped at 0.05 V *vs.* RHE for 10 min to allow the Zn_u_ to completely cover the whole surface of the Ni surface. All of these operations were carried out in a three-electrode electrochemical cell under an Ar atmosphere. The as-prepared Zn_u_ modified Ni_u_/Pd_20_/C@GC was rinsed with deionized water and immersed in a 0.2 M H_2_SO_4_ solution containing 1.0 mM K_2_PtCl_4_ for about 5 min to displace the Zn_u_ with Pt and to obtain Pt atomic layer modified Ni_u_/Pd_20_/C@GC (PtNi/Pd_20_/C@GC).

#### Preparation of Pt_3_Ni(Pt-skin)/Pd_20_/C

The repetitive ZnUPD-Gal process was continually used to introduce the second and the third Pt atomic layers onto the as-prepared PtNi/Pd_20_/C@GC to obtain Pt_3_Ni(Pt-skin)/Pd_20_/C@GC.

#### Preparation of Pt_3_/Pd_20_/C

The repetitive ZnUPD-Gal process continually introduced three Pt atomic layers on Pd_20_/C@GC to obtain Pt_3_/Pd_20_/C@GC.

#### Preparation of (PtNi)_3_/Pd_20_/C

To extend the diversity of the ZnUPD-Gal on Ni_u_, a Pt atomic layer and Ni_u_ repetitively overlapped shell, which was three repeated PtNi layers on a Pd core ((PtNi)_3_/Pd_20_/C), was prepared as a control example. Ni_u_ was electrodeposited on the Pt surface of the as-prepared PtNi/Pd_20_/C@GC from 0.5 M Na_2_SO_4aq_ containing 20 mM NiSO_4_ (Ni_u_/PtNi/Pd_20_/C@GC) at a controlled potential of –0.05 V *vs.* RHE for 20 min to allow the Ni_u_ to completely cover the whole surface of the Pt surface. The Zn_u_ was electrodeposited on the as-prepared Ni_u_/PtNi/Pd_20_/C@GC from 0.5 M Na_2_SO_4aq_ containing 20 mM Zn(ClO_4_)_2_, while controlling the potential at 0.05 V *vs.* RHE for 10 min. Subsequently, the Zn_u_ was replaced by more noble Pt in the Gal process, leading to Pt atomic layer covered Ni_u_/PtNi/Pd_20_/C@GC ((PtNi)_2_/Pd_20_/C@GC). The ZnUPD-Gal on Ni_u_ was used to introduce the third PtNi layer on (PtNi)_2_/Pd_20_/C@GC and obtain (PtNi)_3_/Pd_20_/C@GC.

### Electrochemical characterization of the ORR

A commercial (Pine Instruments) rotating ring-disk electrode (RRDE) was used with a Pt ring and an interchangeable disk. The disk electrode was a GC rod modified by a thin layer of as-prepared electrocatalyst covered by a Nafion polymer thin film used as the working electrode for ORR measurements. The as-prepared working electrode was electrochemically cleaned using a cycling potential between 0.0 and 1.0 V (*vs.* RHE) 10 times in Ar-purged 0.1 M H_2_SO_4_, except for Ni_u_/Pd_20_/C owing to Ni_u_ leaching. The electrochemical surface areas (ECSA) of the electrocatalysts were determined by measuring the areas (charges) under the hydrogen adsorption peaks (H_ad_) (ECSA_H_) and the electro-oxidation of adsorbed carbon monoxide (CO_ad_), otherwise known as CO stripping (ECSA_CO_), respectively, in the cyclic voltammograms. A conversion factor of 0.21 mC cm^–2^ was used to determine ECSA_H_. For CO stripping experiments, CO was adsorbed onto the pre-cleaned electrode by holding the potential at 0.05 V for 10 min in CO saturated 0.1 M HClO_4_ solution. The CO stripping curve was taken after purging with Ar for 30 min. A conversion factor of 0.42 mC cm^–2^ was used to determine ECSA_CO_. The specific ECSA values (sECSA = ECSA/metal loading, m^2^ g^–1^) of the electrocatalysts were evaluated from ECSAH (sECSA_H_) and ECSACO (sECSA_CO_). When considering the ECSA_CO_ contribution from Pt (ECSA_CO(Pt)_ = sECSA_CO_ × the mass of Pt (∼2.97 μg_Pt_ cm^–2^ for Pt_3_Ni(Pt-skin)/Pd_20_/C and Pt_3_/Pd_20_/C)), the Pt content was obtained by measuring the charge associated with Zn_u_ (after correcting for the double layer charging) on Pd_20_/C and assuming that there was a one-to-one ratio between the UPD adlayer and Pt atoms. ORR experiments were performed in oxygen-saturated 0.1 M HClO_4_ aqueous solution. The solution was purged for at least 30 min to ensure oxygen saturation. The ORR electrochemical experiments were conducted in a three-electrode electrochemical cell. Hg/HgSO_4_ (0.5 M H_2_SO_4_) and a Pt wire were used as the reference and counter electrodes, respectively; however, all potentials are quoted with respect to a RHE. The scan rate was 0.01 V s^–1^. A Pine Model AFMSR electrode rotator controlled the electrode rotation for the ORR electrochemical experiments. During measurement of the polarization curves for the ORR on the disk electrode, the Pt ring electrode was potentiostated at 1.1 V *vs.* RHE, a potential where the peroxide oxidation reaction is under pure diffusion control; the collection efficiency, N, for the ring-disk assembly was ∼0.2.

### Accelerated durability test (ADT, linear potential scanning)

The durability of the Pt_3_Ni(Pt-skin)/Pd_20_/C catalyst was investigated in two ADT modes. In the ambient temperature potential polarization, Pt_3_Ni(Pt-skin)/Pd_20_/C@GC was used as the working electrode.

#### Accelerated durability tests related to catalyst lifetime (ADT_s_)

The ADTs were conducted by linear potential sweeping from 0.6 to 1.0 V *vs.* RHE at a scan rate of 50 mV s^–1^ for 20 000 cycles in an O_2_-saturated 0.1 M HClO_4aq_ at room temperature.

#### Accelerated durability test related start-up/shut-down cycles (ADT_on–off_)

The ADT_on–off_ was conducted by linear potential sweeping from 1.0 to 1.5 V *vs.* RHE for 20 000 cycles at a scan rate of 500 mV s^–1^ in O_2_-saturated 0.1 M HClO_4aq_ at room temperature.

## Conflicts of interest

There are no conflicts to declare.

## Supplementary Material

Supplementary information
