# Supplementary material for: High performance layer-by-layer Pt3Ni(Pt-skin)-modified Pd/C for the oxygen reduction reaction
Source: Chem Sci. 2018 Jun 26;9(28):6134–42. doi: 10.1039/c8sc01358f (PMC6053971; doi:10.1039/c8sc01358f)
Supplement: Supplementary file 1 [file SC-009-C8SC01358F-s001.pdf]

Electronic Supplementary Material (ESI) for Chemical Science.
This journal is © The Royal Society of Chemistry 2018

Supporting Information for

High Performance Layer-by-Layer Pt_3Ni(Pt-skin)-modified Pd/C for Oxygen Reduction Reaction

Jing-Fang Huang* and Po-Kai Tseng

Department of Chemistry, National Chung Hsing University, Taichung 402, Taiwan, R.O.C.

*Correspondence to: E-mail: jfh@dragon.nchu.edu.tw

This file includes:

Figures S1-S3

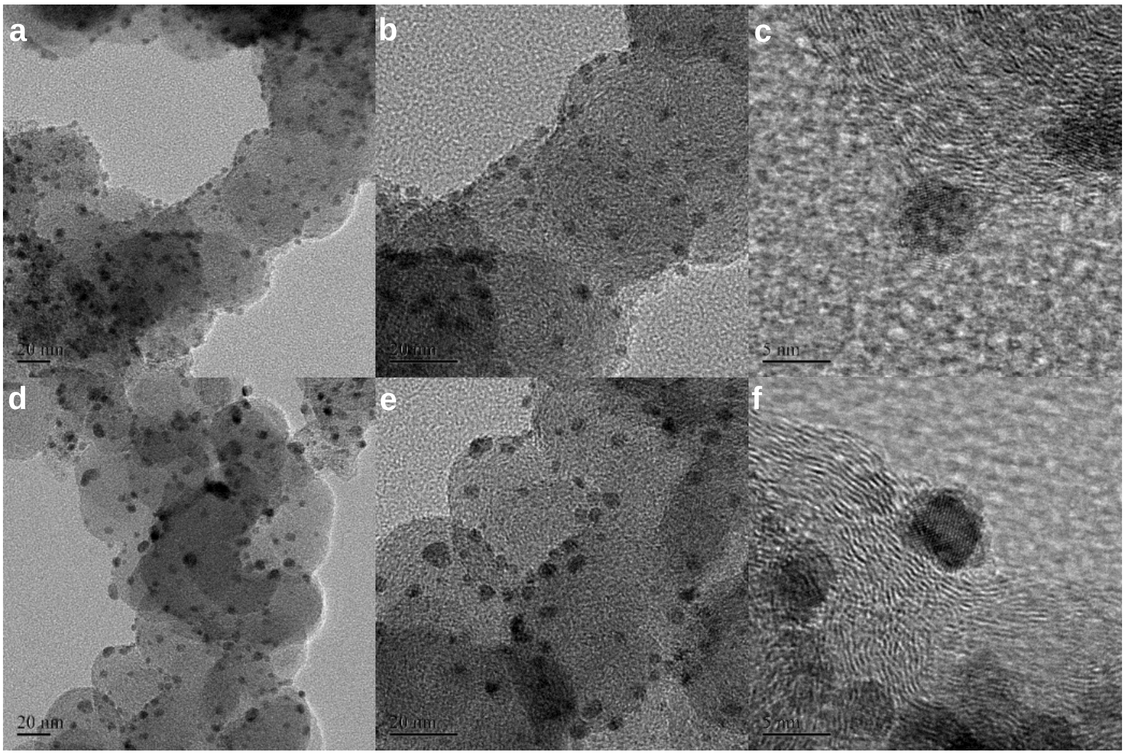

Fig. S1 TEM images of (a)(b)(c) pristine Pd_{20}/C and (d)(e)(f) Pt_3Ni(Pt-skin)/Pd_{20}/C catalysts.

Figure S2.

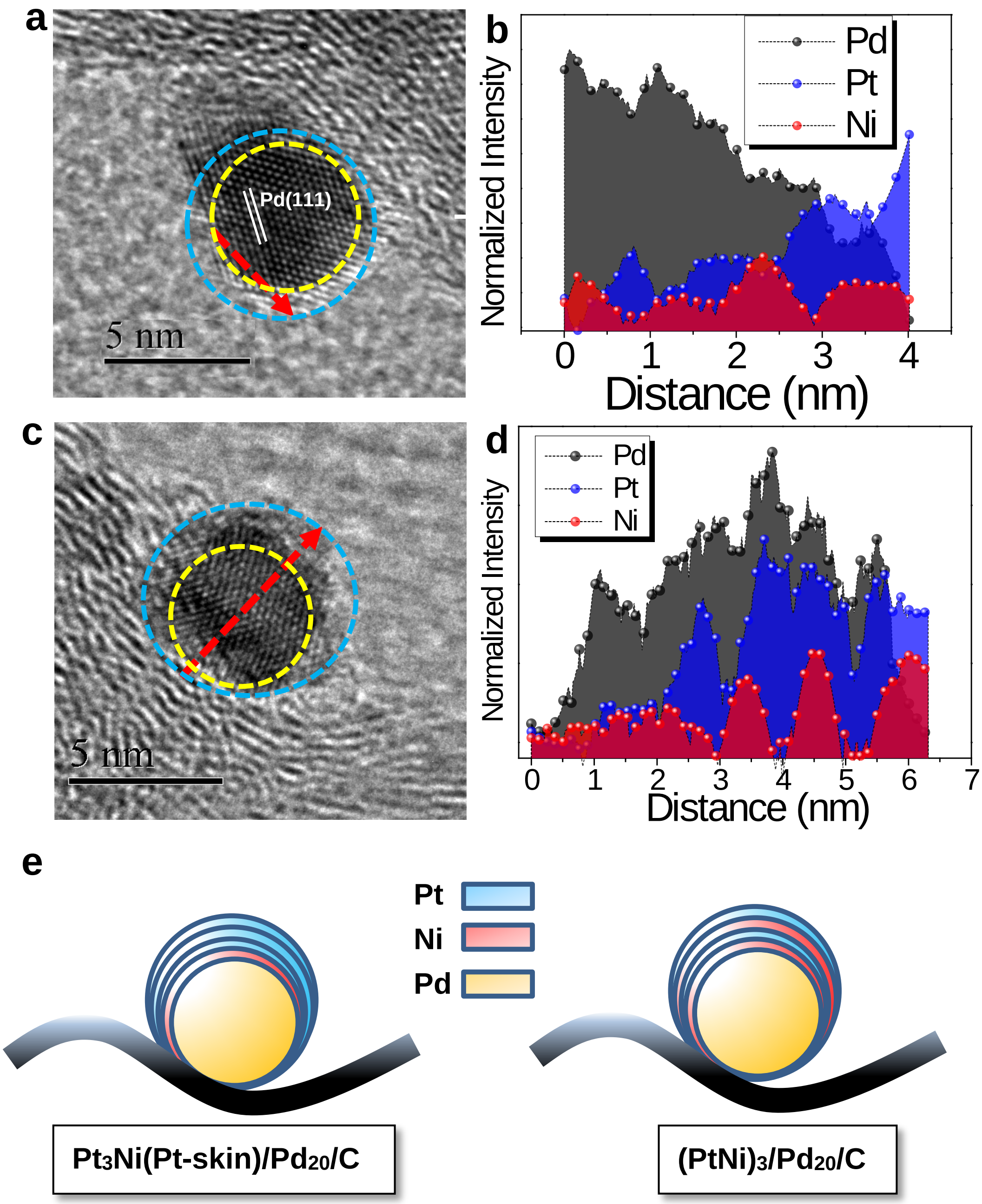

Fig. S2 The representative HRTEM image of (a) a core (Pd)/shell (Pt_3Ni(Pt-skin)) nanoparticle and (c) a core (Pd)/shell $(PtNi)_3$ nanoparticle; (b)(d) the corresponding EDS line-scan profiles along the red dash line shown in (a) and (c). (e) Comparison of layer-by-layer surface configurations for core (Pd)/shell (Pt_3Ni(Pt-skin)) and core (Pd)/shell $(PtNi)_3$ nanoparticles.

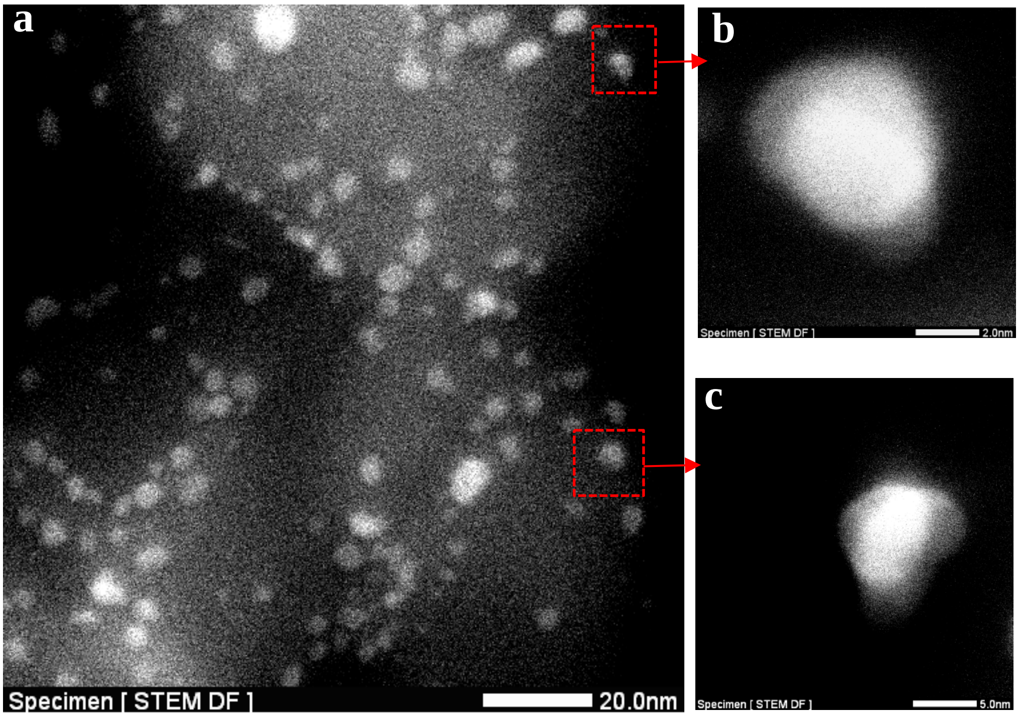

Fig. S3 (a) High-angle annular dark-field scanning TEM (HAADF-STEM) image of a core (Pd)/shell (Pt_3Ni(Pt-skin)) electrocatalyst (b)(c) Corresponding HAADF-STEM images of the regions marked in a. (These images show clear contrast between the shell and the core).
